# Five-Year Follow-Up of Choroidal Metastasis From Lung Adenocarcinoma Harboring Epidermal Growth Factor Receptor (EGFR) Mutation: A Case Report and Literature Review

**DOI:** 10.7759/cureus.66537

**Published:** 2024-08-09

**Authors:** Koki Nakashima, Yoshiki Demura, Toshihiko Tada, Tamotsu Ishizuka

**Affiliations:** 1 Department of Respiratory Medicine, University of Fukui, Fukui, JPN; 2 Department of Respiratory Medicine, Japanese Red Cross Fukui Hospital, Fukui, JPN

**Keywords:** bevacizumab, pemetrexed, egfr-tki, epidermal growth factor receptor (egfr), ocular metastasis, choroidal metastasis, lung cancer

## Abstract

This is a long-term follow-up case report of a 71-year-old man with lung adenocarcinoma and choroidal metastasis harboring an epidermal growth factor receptor mutation. Blurry vision, caused by the choroidal metastasis, improved with first-line treatment with afatinib. Thereafter, osimertinib was administered as a second-line treatment, then chemotherapy containing pemetrexed plus bevacizumab as a third-line treatment. For 61 months, recurrence of choroidal metastasis was absent.

Only a few reports of lung cancer with choroidal metastasis provide long-term follow-up of more than five years. Therefore, the clinical course of this patient may provide some insights for long-term management in such cases.

## Introduction

In lung cancer patients, ocular metastasis is rare [1]. In a large-scale retrospective study, Su et al. reported that ocular metastases from lung cancer are rare in clinical practice with a frequency of about 0.2% [1]. On the other hand, some studies have reported relatively high frequencies, reaching 6-7% [2,3]. For example, Kreusel et al. reported 84 patients in an observational study with metastatic lung cancer and no ocular symptoms who were routinely screened with fundoscopy and ultrasonography. Ocular metastasis was found in six of those patients (7.1%) [2]. Furthermore, Christine et al. reported 89 autopsies of patients who died from lung cancer in a prospective study and found ocular metastasis in six of those patients (6.7%) [3]. The differences in these frequency results suggest that a relatively large number of lung cancer patients with ocular metastasis may not be diagnosed before death in clinical practice. Since ocular metastasis may be more common than previously reported [1], clinicians should consider this when managing such patients.

Recently, an increasing number of reports have shown the efficacy of epidermal growth factor receptor (EGFR)-tyrosine kinase inhibitors (TKIs) for patients with non-small cell lung cancer (NSCLC) harboring an EGFR mutation with ocular metastasis [4-14]. However, there are few reports of such cases with long-term follow-up. Therefore, information showing long-term management for such cases is necessary.

Herein, we report a case of NSCLC harboring an EGFR mutation with choroidal metastasis and more than five years of follow-up, along with a review of the literature.

## Case presentation

**Figure 1:**
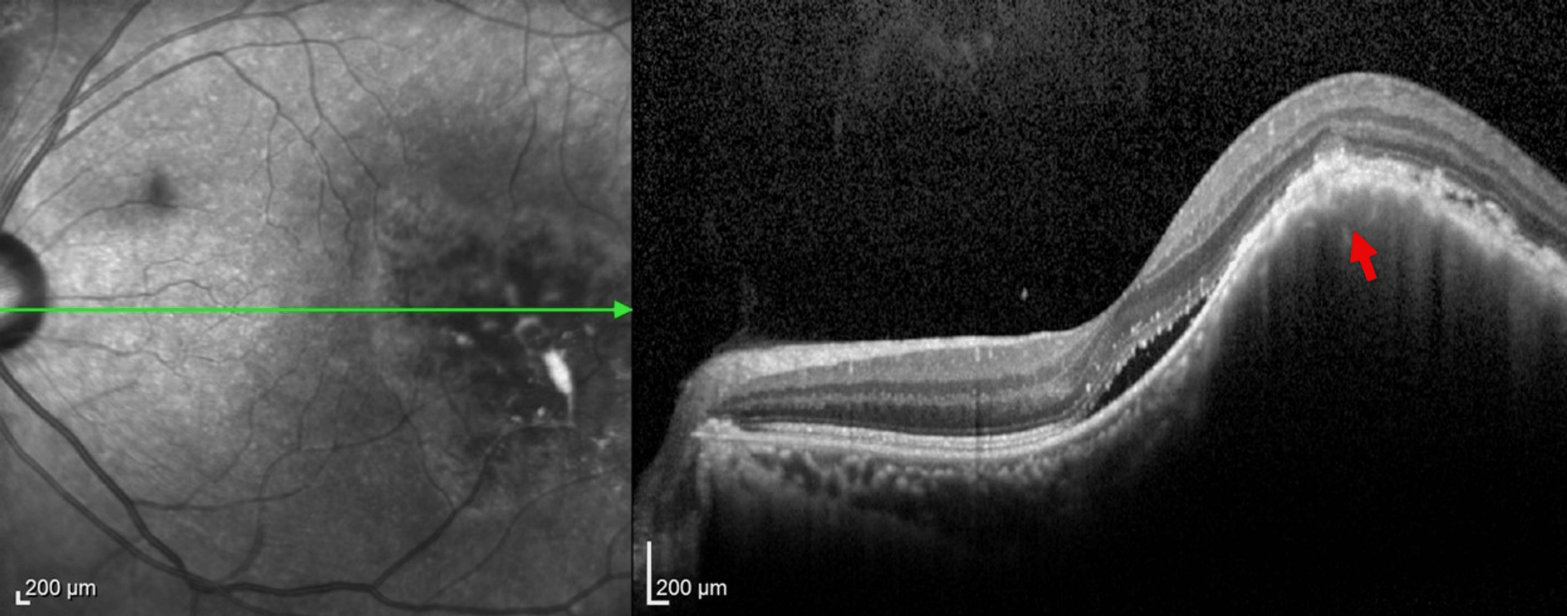
Optical coherence tomography at the first visit Optical coherence tomography at the first visit to an ophthalmologist shows a choroidal mass in the left eye (red arrow).

**Figure 2:**
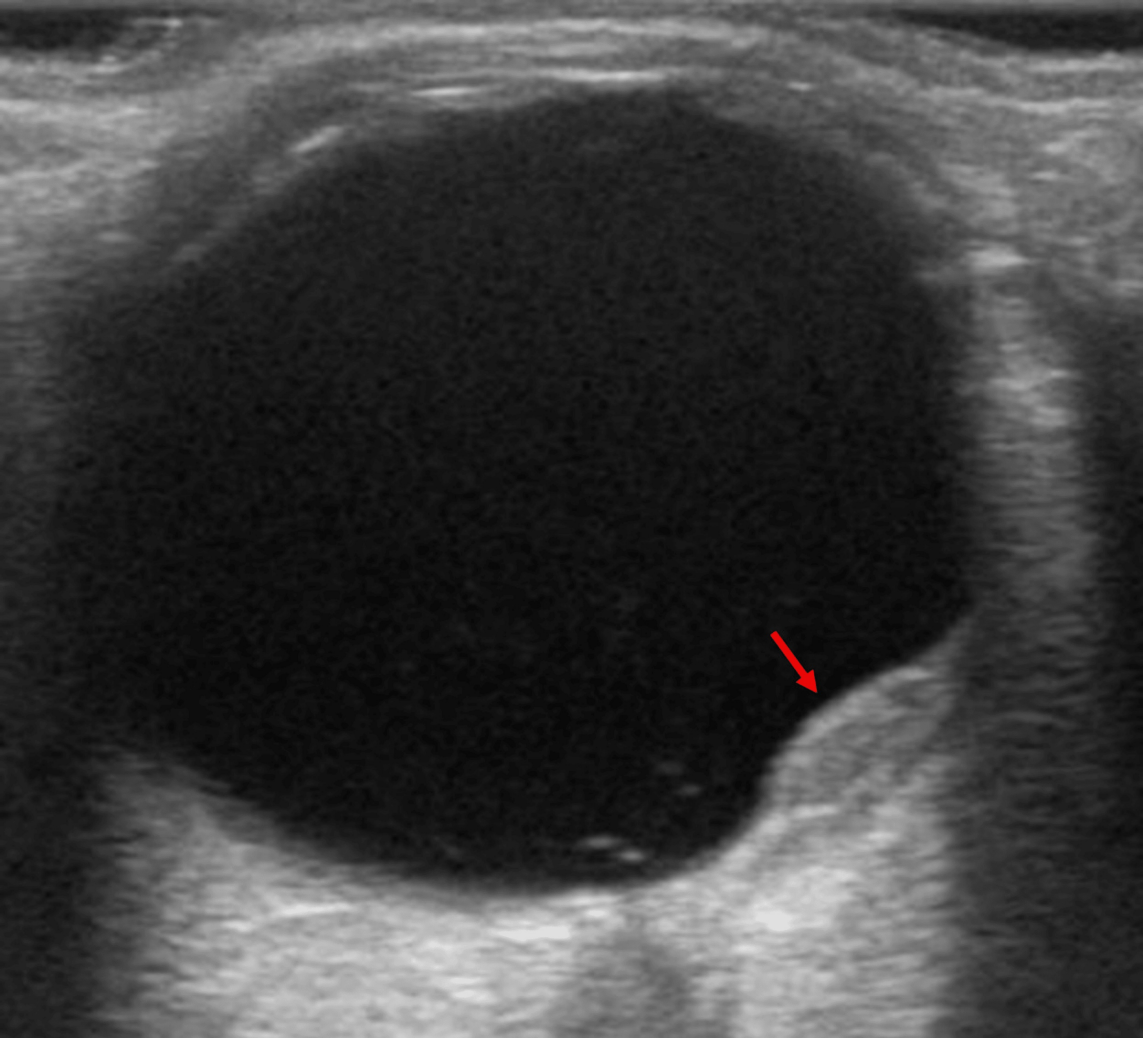
Ultrasonography of the left eye Ultrasonography of the left eye also shows the choroidal mass lesion (red arrow).

A 71-year-old man with a 50-year smoking history visited an ophthalmologist for two months, complaining of blurry vision. Optical coherence tomography (OCT) revealed a choroidal mass in the left eye, which was suspected to have metastasized (Figure 1). Ultrasonography of the left eye also detected the lesion, leading to a referral to our hospital for a more detailed examination (Figure 2).

Computed tomography revealed a primary tumor in the right upper lung and multiple hilar and mediastinal lymph nodes. Magnetic resonance imaging detected multiple brain micro-metastases. An endobronchial ultrasound-guided transbronchial needle aspiration of the #7 lymph node was performed, and pathological analysis indicated adenocarcinoma. Polymerase chain reaction detected a mutation in EGFR (exon 21 L858R); therefore, the patient was diagnosed with stage IV lung adenocarcinoma harboring an EGFR mutation.

**Figure 3:**
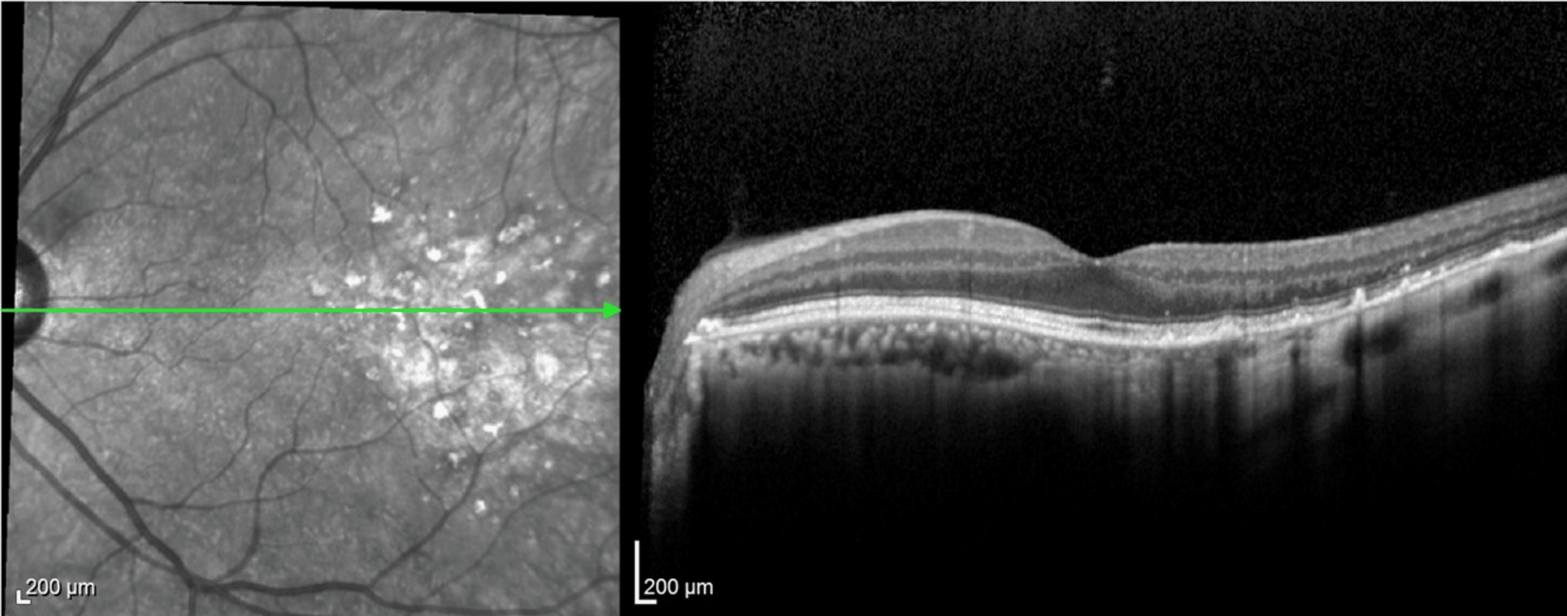
Optical coherence tomography after three months since initiation of afatinib Optical coherence tomography after three months since initiation of afatinib shows no detection of the mass.

The patient received afatinib as a first-line treatment. Subsequently, the choroidal metastasis and multiple brain micro-metastasis disappeared with complete improvement of the blurry vision (Figure 3). Other neoplastic lesions had a partial response. Ophthalmologic examinations continued every three months thereafter. We detected the progression of the primary tumor 18 months after starting afatinib. Re-biopsy of the primary tumor detected T790M, leading to the initiation of osimertinib as a second-line treatment. After 18 months, it was revealed that the primary tumor and brain metastases progressed. After whole-brain radiation therapy, a third-line treatment with carboplatin, pemetrexed, and bevacizumab was administered, followed by maintenance therapy with pemetrexed and bevacizumab. After 22 months since the initiation of the third-line treatment, the maintenance therapy was discontinued due to a gradual decline in food intake and performance status, even though the choroidal metastasis remained a complete response (CR) and other neoplastic lesions had reduced in size. Ultimately, the patient died three months after treatment discontinuation (61 months after diagnosis), with no recurrence of the choroidal metastasis.

## Discussion

This report describes a case of choroidal metastasis from NSCLC harboring an EGFR mutation with long-term follow-up. This case, to the best of our knowledge, had the longest follow-up compared to previous reports [4-14]. As a result, this clinical course may provide some important insights for the long-term management of such patients. Details of previous NSCLC cases with an EGFR mutation and choroidal metastasis, treated with an EGFR-TKI as a first-line treatment, are provided in Table 1.

**Table 1:** Details of previous NSCLC cases harboring an EGFR mutation with choroidal metastasis and treated with an EGFR-TKI as a first-line treatment EGFR-TKI: epidermal growth factor receptor-tyrosine kinase inhibitor (-) : currently alive

	Age	Sex	Ocular symptoms	EGFR mutations	1st-line EGFR-TKI	Outcome of ocular symptoms	Overall survival (months)	References
1	62	Male	Blurry vision, partial visual field loss	Exon 19 deletion	Osimertinib	Improved	20-	[4]
2	66	Male	Visual acuity loss	Exon 19 deletion	Osimertinib	Improved	Unknown	[5]
3	68	Male	Choroidal mass without any symptoms (routine eye examination)	Exon 19 deletion	Osimertinib	Improved	17-	[6]
4	63	Male	Blurry vision	Unknown	Osimertinib	Improved	9-	[7]
5	41	Female	Visual disturbance, blurry vision	Unknown	Erlotinib	Improved	30-	[8]
6	41	Male	Vision loss, pain, red eye	L858R	Osimertinib	Unknown	Unknown	[9]
7	60s	Male	Myodesopsia, visual acuity loss	Exon 19 deletion	Afatinib	Unknown	25-	[10]
8	60s	Female	Blurry vision	Exon 19 deletion	Afatinib	Unknown	17	[10]
9	50s	Female	Visual acuity loss	L858R	Erlotinib	Unknown	27	[10]
10	40s	Female	Blurry vision	L858R	Erlotinib	Unknown	6	[10]
11	50s	Male	Choroidal mass without any symptoms (MRI-based diagnosis)	Exon 19 deletion	Erlotinib	Unknown	36	[10]
12	49	Male	Blurry vision, metamorphopsia	Exon 19 deletion	Afatinib	Improved	Unknown	[11]
13	56	Female	Visual acuity loss	Exon 19 deletion	Gefitinib	Improved	6-	[12]
14	55	Male	Visual acuity loss	Unknown	Afatinib	Improved	12-	[13]
15	52	Female	Myodesopsia, blurry vision, narrowed visual field, visual acuity loss	Exon 19 deletion	Gefitinib	Improved	5-	[14]
Our case	71	Male	Blurry vision	L858R	Afatinib	Improved	61	

Firstly, it is important to detect driver oncogenes in patients with choroidal metastasis from NSCLC. Driver oncogenes, such as EGFR, should be appropriately tested in NSCLC patients with ocular metastasis. According to previous cases of NSCLC harboring an EGFR mutation with choroidal metastasis treated with EGFR-TKI as a first-line treatment [4-14], ocular symptoms can improve with EGFR-TKI in most cases, although some reports do not provide details of the therapeutic effects. Furthermore, Maller et al. also reported that lung cancer patients with intraocular metastases can achieve long-term survival when a molecularly targeted therapy is feasible [15].

Another insight from this case report is that subsequent treatment combining pemetrexed and bevacizumab after failure of EGFR-TKI may be beneficial in long-term maintenance of CR in ocular metastasis. Since EGFR-TKI led ocular metastasis to CR in the present case, the efficacy of the subsequent treatment combining pemetrexed and bevacizumab on ocular metastasis is difficult to determine. However, the treatment response of CR in ocular metastasis was only an imaging evaluation, not a pathological evaluation. Therefore, the subsequent treatment combining pemetrexed and bevacizumab may have provided some benefits on long-term maintaining CR in ocular metastasis, even though few reports have provided information on subsequent treatment in this setting. Riess et al. reported that chemotherapy containing pemetrexed and bevacizumab was effective in three patients with NSCLC and ocular metastasis positive for driver oncogenes (two patients with an EGFR mutation and one with an ALK fusion gene), although all of them received chemotherapy containing pemetrexed and bevacizumab as a first-line treatment [16]. In the report, it was hypothesized that pemetrexed might be useful in treating ocular metastasis. Interestingly, Kim et al. reported that combing intravitreal bevacizumab and EGFR-TKI had a clinical benefit in an NSCLC patient with choroidal metastasis [17]. Inhibition of vascular endothelial growth factor (VEGF) may also be effective against ocular metastasis. However, since VEGF inhibitors have risks of bleeding, further studies are needed to evaluate the efficacy and safety of chemotherapy containing pemetrexed and bevacizumab in patients with ocular metastasis from NSCLC.

## Conclusions

In conclusion, since ocular metastasis may be more common in NSCLC patients than previously reported, it is important to elucidate management for such cases. Since EGFR-TKIs may be effective for long-term management, it is important to detect driver oncogenes in patients with ocular metastasis from NSCLC.
